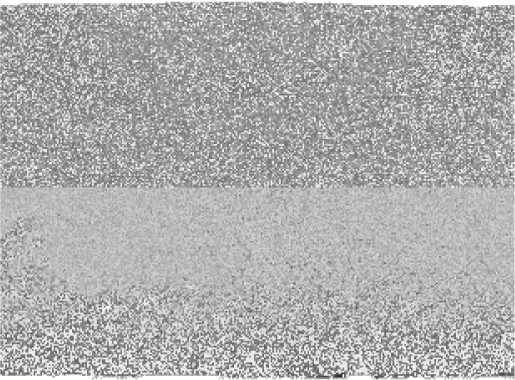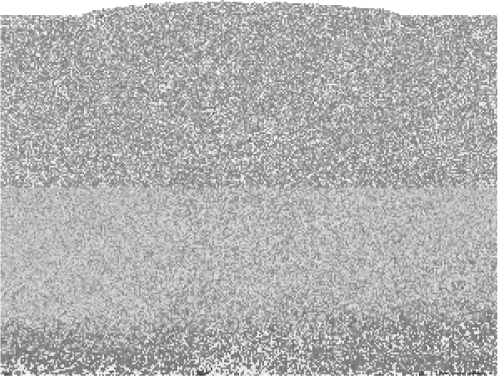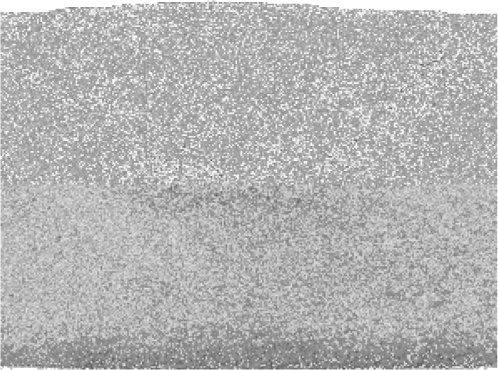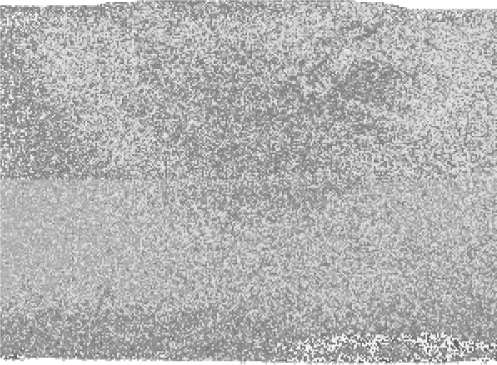# Exploring Infrared Sensoring for Real Time Welding Defects Monitoring in GTAW

**DOI:** 10.3390/s100605962

**Published:** 2010-06-14

**Authors:** Sadek C. A. Alfaro, Fernand Díaz Franco

**Affiliations:** Automation and Control Group, Departamento de Engenharia Mecânica/Mecatrônica, Faculdade de Tecnologia, The Brasilia University, Brasília–DF, CEP: 70910-900, Brazil

**Keywords:** Gas Tungsten Arc Welding, welding monitoring, infrared, statistic methods

## Abstract

This paper presents an evaluation of an infrared sensor for monitoring the welding pool temperature in a Gas Tungsten Arc Welding (GTAW) process. The purpose of the study is to develop a real time system control. It is known that the arc welding pool temperature is related to the weld penetration depth; therefore, by monitoring the temperature, the arc pool temperature and penetration depth are also monitored. Various experiments were performed; in some of them the current was varied and the temperature changes were registered, in others, defects were induced throughout the path of the weld bead for a fixed current. These simulated defects resulted in abrupt changes in the average temperature values, thus providing an indication of the presence of a defect. The data has been registered with an acquisition card. To identify defects in the samples under infrared emissions, the timing series were analyzed through graphics and statistic methods. The selection of this technique demonstrates the potential for infrared emission as a welding monitoring parameter sensor.

## 1. Introduction

Technology advancements seek to meet the demands for quality and performance through product improvements and cost reductions. An important area of research is the optimization of applications related to welding and the resultant cost reduction. The use of non destructive tests and defect repair are slow processes. To avoid this, online monitoring and control of the welding process can favor the correction and reduction of many defects before the solidification of the melted/fused metal, reducing the production time and cost.

With continuing advancements in digital and sensor technology, new methods with relatively high accuracy and quick response time for identification of perturbations during the welding process have become possible. Arc position, part placement variations, surface contaminations and joint penetration are key variables that must be controlled to insure satisfactory weld production [1].

The techniques related to welding process optimization are based on experimental methodologies. These techniques are strongly related to experimental tests and seek to establish relations between the welding parameters and welding bead geometry. The introduction of close or adaptive control to welding processes must be done by monitoring a variable or set of variables which can identify a process disturbance. For each practical implementation of an adaptive system to a welding process one should identify the “envelope” or the set of monitoring variables. These variables must be used as a reference value in the process control, making the system control start with a parameter adjustment (welding current, voltage, *etc*.) to guarantee bead characteristics close to desirable values. The welding parameters vary in accordance to base material, type of chosen process, plate dimensions and welding bead geometry, so the adjustment of the reference value of a monitored variable will depend on the establishment of a set of optimized parameters which provide a welding bead with desirable specifications.

Researches related to adaptive systems for welding seek the improvement of welding bead geometry with direct (if based on monitoring sensors) or indirect monitoring techniques. The indirect monitoring systems are the more used, looking to link elements such as welding pool vibrations, superficial temperature distribution and acoustic emissions to size, geometry or welding pool depth [2]. According to Hong, the most used approaches in welding control are infrared monitoring, acoustic monitoring, welding pool vibrations and welding pool depression monitoring [3].

Aiming to optimize human analysis during the defect identification process, many researches were conducted to develop alternative techniques for automatic identification of defects considering different classes of signals such as plasma spectrum [4], ultrasonic [5], computer vision [6], *etc.*

Arc welding is intrinsically a thermal processing method. To this end, infrared sensing is a natural choice for weld process monitoring. Infrared sensing is a non-contact measurement of the emissions in the infrared portion of the electromagnetic spectrum.

## 2. Infrared Monitoring

During the welding process, the high temperature associated with the arc and appropriate thermophysical properties such as thermal diffusivity cause strong spatial temperature gradients in the region of the weld pool. Convection in the weld pool, the shape of the weld pool and the heat transfer in both the solid and liquid metal determine the temperature distributions in the plate and on the surface. For an ideal weld with stable conditions, these surface temperatures should present repeatable and regular patterns. Perturbations in welding penetration should be clearly identifiable from variations in the surface temperature distribution [7].

Infrared emissions indicate the heat content of the weld. For example, deeper penetration tends to correlate with increased heat input (caused by higher current or slower weld speed). Greater heat input results in higher temperatures and increased infrared emissions [8]. The temperature may be monitored by a pyrometer, but depend on the kind of sensor is using, due to the slow response time of the system and the presence of an intense thermal signal from the welding focused area (saturation problems). According to Sanders, a better indicator is the infrared energy emitted by the weld, including both the contributions from the weld pool and plasma.

It is necessary to carry out the temperature measurement with a sensor that doesn’t introduce defects during the welding process. It is for this reason that non contact temperature sensors are more suitable. An infrared thermometer measures temperature by detecting the infrared energy emitted by all materials which are at temperatures above absolute zero, (0 Kelvin).

The infrared monitoring techniques for weld pool are: area scanning and point monitoring. Area scanning provides a bidimensional view of the surface temperature distribution profile, making possible a complete analysis of the heat transfer process during welding [1,7]. Considering that we are dealing with bidimensional images, the application of area scanning demands a better computational structure (hardware and software), requiring a longer processing time [9]. On the other hand, the point monitoring technique demands little computational structure, requiring a shorter processing time, which makes it more appropriate for controlling in real time [10,11]. A recent study presented the adaptive control of welding through the infrared monitoring of the weld pool using point sensors [12]. The most basic design consists of a lens to focus the infrared (IR) energy on to a detector, which converts the energy to an electrical signal. This configuration facilitates temperature measurement from a distance without contact with the object to be measured [13].

**Figure 1.**
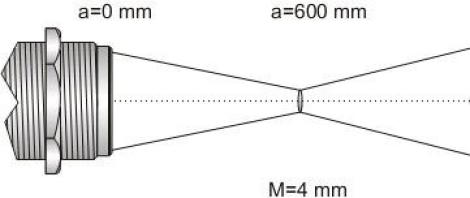
TL-S-25 Infrared Sensor focus (Calex Electronics Ltd).

To make a correct measurement with this class of sensors, it is necessary to focalize the area that is going to be measured; this is possible by knowing the focal distance of the lens. Figure 1 shows a focal distance for one infrared sensor. In this case, a focal distance has a length of 600 mm and a radius of 4 mm (waist radius).

## 3. Failure Detection

This study compares two algorithms for defect detection. The first one used is the conventional Kalman filter together with the Mahalanobis distance calculus to evaluate the presence of failures. In the second, the linear regression Kalman filter–LRKF and the generalized likelihood ratio test [14] are used to determine the distance between the autoregressive model and the signal read.

### 3.1. Change Detection

**Figure 2.**
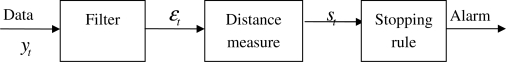
Basic change detection flux.

This is a statistical technique that can detect abrupt changes in signals. Since welding is a stochastic process [15], some properties and algorithms can be applied. It consists basically on the flux of Figure 2.

Under certain model assumptions, adaptive filters take the measured signals and transform them to a sequence of residuals that results in a white before the change occurs [16]. If there is no change in the system and the model is correct, then the residuals are a sequence of independent variables with zero mean and known variance. When a change occurs, it can reflect on some ariation in the mean, variance or both values that makes the residuals greater. The main point is to establish how great is this value to assume that a change had occurred. The statistical test decides whether the deviation is significant or not. The evaluation is usually made on four situations, change in the mean, change in the variance, change in correlation and change in signal correlation. In this work the evaluation was made on the mean and it is based on the residuals.

The stopping rule is based on the distance measurement. Many change detection algorithms make a decision between two hypotheses:

(1) $$\begin{array}{l} H_{0}:E(s_{t})=0,\\ H_{1}:E(s_{t})>0 \end{array}$$

This rule is achieved by the value calculated by the low-pass filter *s_t_* and compares to a threshold. If the value is greater, an alarm is set.

### 3.2. Kalman Filter

A simple description of the infrared signal behavior as a discrete temporal series can be made in terms of an autoregressive model (AR) of order m. The present sample value is represented by the linear combination of m past samples incremented by a parameter of uncertainty. For a temperature registry of a component z[t], According to Pollock [17] the model AR of order m is given by Equation (2):

(2) $$Z[t]=\sum_{i=1}^{m}a_{i}\;z[t-i]+\varepsilon [t]$$

where a_i_ = {a1, . . ., am} are the coefficients of model AR and ε[t] is the noise component to represent the inaccuracy of the signal reading during welding. It is supposed that the sequence ε[1 : t] = {ε [1], ..., ε [t]} is independent and identically distributed (i.i.d) Gaussian with mean E{ε [n]} = 0, variance E{(ε[n])2} = σ2.

From the observation of different statistic characteristics in the noise residues and the presence of defect in a model AR of order m, it is possible to establish a recursive estimation system using a stochastic filtration technique to observe and track the temperature interval in which the gaussianity of the sequence is preserved. One of these tools is the Kalman filter. The state vector is given by Equation (3) [18]:

(3) x[k]=A[k]x[k−1]+w[k]

where x[k] is the state vector of dimension n, A[k] is a square matrix of state transition, w[k] is a sequence of dimension n of Gaussian white noise of null mean. The observation model is given by Equation (4):

(4) z[k]=H[k]x[k]+v[k]

in which z[k] is the observation vector of dimension m, H[k] is the measuring matrix and v[k] represents Gaussian white noise of null mean. It is supposed that the w and v processes are non-correlated and also:

(5) $$\begin{array}{l} \begin{array}{r} E\{w[k]w{\left[i\right]}^{T}\}=\left\{ \begin{array}{lll} Q[k], & \mathrm{if} & k=i\\ 0, & \mathrm{if} & k\neq i \end{array}\right.\\ E\{v[k]v{\left[i\right]}^{T}\}=\left\{ \begin{array}{lll} R[k], & \mathrm{if} & k=i\\ 0, & \mathrm{if} & k\neq i \end{array}\right. \end{array} \end{array}$$

In this system, the initial state *x*[0] is a random Gaussian variable of mean *x̂*[0] and matrix of covariance P[0]. *x*[0] is supposedly non-correlated to the w and v processes. The basic problem of the Kalman filter is to obtain an estimation *x̂*[*k*|*k*] of *x*[*k*] from the measurement {z[1], z[2], . . ., z[k]}, in order to minimize a metric of mean square error. This metric is given by the trace of the *a posteriori* error covariance matrix as presented in Equation (6):

(6) $$P[k|k]=E\left\{(x[k]-\hat{x}[k|k]){\left(x[k]-\hat{x}[k|k]\right)}^{T}\right\}$$

Fortunately, this estimation problem presents a recursive solution. This solution is given in two stages. First there is a prediction stage (between observation Equation (7, 8)), in which:

(7) $$\hat{x}[k|k-1]=A[k]\hat{x}[k-1|k-1]$$

(8) $$P[k|k-1]=A[k]P[k-1|k-1]A{\left[k\right]}^{T}+Q[k]$$

Then, there is the correction stage in which the actual observation is used to correct the prediction *x̂*[*x*|*k* – 1]:

(9) $$\hat{x}[k|k]=\hat{x}[k|k-1]+H[k](Z[k]-H[k]\hat{x}[k|k-1])$$

(10) P[k|k]=P[k|k−1]−K[k]H[k]P[k|k−1]

in which:

(11) $$K[k]=P[k{|k-1]H{\left[k\right]}^{T}\left[H[k]P[k|k-1]H{\left[k\right]}^{T}+R[k]\right]}^{-1}$$

is named Kalman gain.

The main idea concerning the defect identification is related to the use of a statistic test that, jointly with stochastic filtration, verifies if the infrared samples properties are related to the estimation of the model AR given by the Kalman filter. If the test fails, it is supposed that the actual sample correlates with the presence of defect.

The comparison between the infrared signal sample and the recursive estimation consists in the Chi-square probabilistic hypothesis through the Mahalanobis distance [19]. Such a distance is a natural measurement that indicates, in a probabilistic sense, how much of the registry of the actual sample is compatible with the estimated infrared signal model, estimated by the Kalman filter.

## 4. Experimental Procedure

All the experiments in this study were conducted using a gas tungsten arc welding (GTAW) bead on a plate. The plates used were SAE 1020 6.35 mm thick 30 cm × 20 cm in size. The surfaces of these edges were then prepared for welding using standard preparation techniques. Other important parameters for the experiments are given in Table 1.

Welding was performed with an IMC Inversal 450 power source. The power source was configured to operate on remote control. To avoid the arc from changing position when the torch is performing conventional welding moves, a positioning table that moves the plate without moving the torch during the experiment was used. The positioning table was controlled by a microcontroller communicating with a computer using the RS-232 protocol. The computer sends to the microcontroller the welding speed, start and end signal.

The infrared radiation was captured by an infrared sensor model TL-S-25 that gives a current sign between 4–20 mA which is proportional to the registered temperature (measuring Range 800–2,500 °C). An infrared filter was used to prevent saturating the infrared sensor with a weld current above 150A. Finally, the sensor current was converted to voltage using a current/voltage converter.

**Figure 3.**
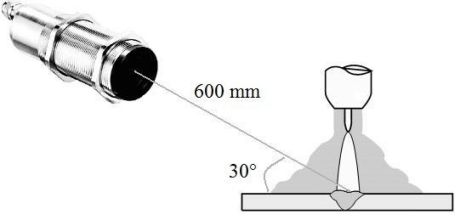
Position measurement for the infrared sensor.

To locate the position of the sensor correctly (arc welding and weld pool), the TL-S-25 pattern provides a tool for localizing the best place for the temperature measurement. This tool is a laser incorporated into a sensor, which shows the focus; for this pattern the focus localization is 600 mm. Figure 3 shows how the sensor was positioned.

For measuring the welding current and comparing it with infrared signal, a current clamp model i1010 from FLUKE was used. It measures currents without breaking the electric circuit using the Hall effect. The welding current and infrared signals are digitalized by a model PCI703S-16 (Eagle Technology) acquisition board.

The power source can be controlled by a computer that generates an analog signal between −5 and +5 volts and two digital TTL signs. If the analog signal presents a value within this region, the power source will supply a current between −450 and +450 A; while the TTL signs control the opening/closing of the protection gas and the arc on/off.

**Figure 4.**
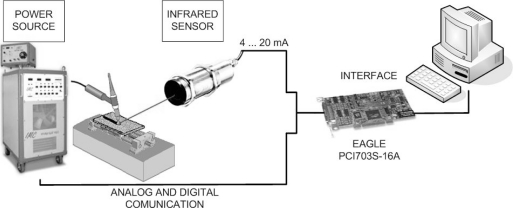
Experimental scheme. Block diagram of the data-acquisition and control system.

A software program was developed in LabVIEW to control the power source and to register the chosen parameters (current, voltage and temperature). An open control was implemented to set the welding parameters. The current, the voltage and the temperature signals were digitized using an acquisition data board (PCI703S-16). The sampling frequency was 15,000 samples per second for each channel. The variables observed in function of length allow a better visualization of what happened during the process. Using the welding speed and time process, the graph of the variables in function of length can easily be obtained. Experiments were carried out with a fixed current and defects throughout the path of the weld bead were artificially applied. The experimental setup used to perform the welds is shown in Figure 4.

## 5. Results and Discussion

The Results and Discussion are divided into two sections. In the first, the weld infrared signal use is evaluated. Comparisons between weld current stability signal and infrared radiations are done. The second part demonstrates the use of the infrared signal for defect detection, according to the model presented.

### 5.1. Infrared Emission Evaluation

An infrared filter was used to prevent sensor saturation and it changed the sensor characteristic curve. As it was not relevant to know the absolute temperature value during the welding, but it was necessary to know the infrared emissions variations, the graphs show relative values for infrared emissions and weld current.

**Figure 5.**
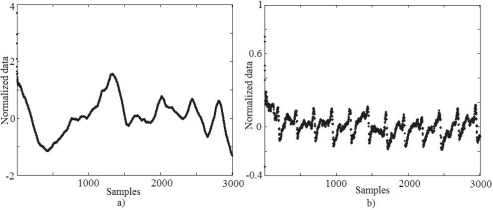
Normalized signals comparison. (a) Infrared signal. (b) Weld current.

The aim of the first experiment was to compare the informations given by the infrared and current sensors. The Figure 5 shows the current and infrared normalized signals produced during the experiment without the presence of defects. Given that the welding machine works as a current source, the current signal that was read is practically invariant in time. The information provided by the current clamp contrasts with the infrared sensor information that shows an important variation in its signal even with a constant current. The mean value of the infrared signal or DC (direct current) level of the signal is related to weld penetration depth, while AC (alternating current) portions of the output can be correlated with surface irregularities and part misalignment or contamination. Thus, the information given by the infrared sensor provides more information than the current weld, allowing infrared sensor use to evaluate the quality of the weld and also the detection of some defects.

It is necessary to verify if the infrared signal corresponds to the weld current variations, in order to relate the infrared radiation intensity with weld penetration depth.

**Figure 6.**
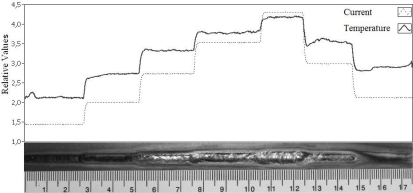
Temperature behavior with currents varied.

Figure 6 shows an experiment in which the current was altered along the process. These variations aim to show the temperature dependency to the current alterations, given that the current variations can produce defects during the welding. It is observed that the current increase corresponds to a proportional increase in the value registered by the sensor. As expected, the same is observed when the current value is reduced.

To correlate the infrared signal and the weld penetration depth, the piece was submitted to a metallographic test. Table 2 compares the penetration depth between them. Greater values of the infrared signal correspond to a greater penetration depth.

### 5.2. Defect Detection

**Figure 7.**
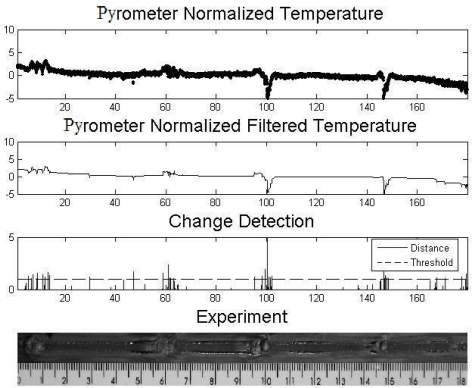
Plate with iron defects.

**Figure 8.**
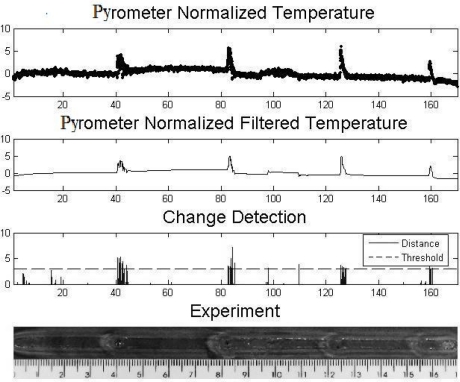
Plate with water defects and change detection analysis.

**Figure 9.**
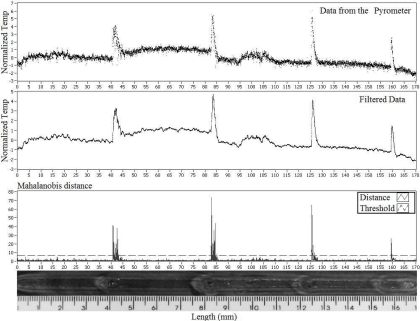
Current Plate with water defects and Mahalanobis distance.

Figures 7, 8 and 9 show experiments in which some defects were incorporated into the path of the weld bead, with the purpose of observing the temperature behavior. The temperature presents variations in the nearness of the defects and envelopes them. The first two experiments consisted of metallic inclusion and in the third one water was sprayed. 1 metal 2 water described

Figure 7 shows the result of an experiment in which defects were introduced by dispersing iron pieces along the length 7, 60, 100, 149 mm. The Figure shows the signal read by the sensor; we also see there the filtered signal according to LRKF. We can observe the values produced by the algorithm of change detection, which together with the Stopping rule provides defects detection. It is observed that all the defects reached the minimum value of the stopping rule, but some other regions where the presence of defects was detected are also observed. Along the length at 46 mm, a change on the width of the bead is observed, demonstrating that there was an involuntary change in some of the power source parameters that caused the anomaly.

Figures 8 and 9 show an experiment in which the defects were introduced through the presence of water during the welding process. Figure 8 shows an analysis done with the generalized likelihood ratio test, and Figure 9 shows an analysis done according to the Mahalanobis distance. In Figure 8 we observe four clearly detected defects, it is observed that the last two defects remained on the limit of the Stopping Rule and two more anomalies were detected around 108 and 110 mm. In just one of them (108 mm) a variation in the form of a bead is observed.

Figure 9 shows an analysis according to the Mahalanobis distance. It is observed that the distance to the region where there is no presence of defects (constant temperature) is located below the threshold proposed by the statistic test. During the presence of the defect, the residue between the real sample Z[k] and the sample estimated by the AR coefficient increases at such a rate that the distance Z[k] surpasses the established threshold where the defect presence is verified. We should also note that this test could not detect the anomalies presented around 105 mm.

## 6. Conclusions

The exploration of infrared sensoring as an indicator for defect detection during the GTAW process was discussed in this paper. The relationship between the infrared signal and the weld penetration depth was shown. Infrared weld pool monitoring in the GTAW process provides information about penetration depth. It also shows that infrared signal variations in DC are related to weld penetration depth, while AC portions of the output can be correlated with surface irregularities.

Together with a change detection algorithm, the system monitors the residual of the regression algorithm, looking for changes in the mean. The proposed method maintains a regression model where residuals are filtered by a Kalman filter. A Mahalanobis distance algorithm monitors significant changes in the output of the Kalman filter. The Kalman filter has a good performance in detecting real changes from noisy data. The simplicity of the proposed algorithm permits its implementation in systems for monitoring, detection and localization of events in real time.

## Figures and Tables

**Table 1.** Welding Conditions.

Welding Speed (Positioning Table)	2.5 mm/s
Shielding Gas	Argon 10 L/min
Current	90 A DC
Electrode	Negative EWTh-2, 1.6 mm
Stand-Off	5 mm

**Table 2.** Relation between infrared signal and weld penetration depth under different currents.

**Current (A)**	90	155	180	130
**Infrared read (V)**	3.31	4.01	4.27	3.68
**Image**